# A novel m7G-related lncRNA risk model for predicting prognosis and evaluating the tumor immune microenvironment in colon carcinoma

**DOI:** 10.3389/fonc.2022.934928

**Published:** 2022-08-04

**Authors:** Sheng Yang, Jiahui Zhou, Zhihao Chen, Qingyang Sun, Dongsheng Zhang, Yifei Feng, Xiaowei Wang, Yueming Sun

**Affiliations:** ^1^ First Clinical Medical College, Nanjing Medical University, Nanjing, China; ^2^ Department of General Surgery, First Affiliated Hospital, Nanjing Medical University, Nanjing, China

**Keywords:** colon carcinoma, N7-methylguanosine(m7G), long noncoding RNA(LncRNA), risk model, tumor immune microenvironment

## Abstract

N7-Methylguanosine (m7G) modifications are a common type of posttranscriptional RNA modifications. Its function in the tumor microenvironment (TME) has garnered widespread focus in the past few years. Long non-coding RNAs (lncRNAs) played an essential part in tumor development and are closely associated with the tumor immune microenvironment. In this study, we employed a comprehensive bioinformatics approach to develop an m7G-associated lncRNA prognostic model based on the colon adenocarcinoma (COAD) database from The Cancer Genome Atlas (TCGA) database. Pearson’s correlation analysis was performed to identify m7G-related lncRNAs. Differential gene expression analysis was used to screen lncRNAs. Then, we gained 88 differentially expressed m7G-related lncRNAs. Univariate Cox analysis and Lasso regression analysis were performed to build an eight-m7G-related-lncRNA (ELFN1-AS1, GABPB1-AS1, SNHG7, GS1-124K5.4, ZEB1-AS1, PCAT6, C1RL-AS1, MCM3AP-AS1) risk model. Consensus clustering analysis was applied to identify the m7G-related lncRNA subtypes. We also verified the risk prediction effect of a gene signature in the GSE17536 test set (177 patients). A nomogram was constructed to predict overall survival rates. Furthermore, we analyzed differentially expressed genes (DEGs) between high-risk and low-risk groups. Gene Ontology (GO) analysis and Kyoto Encyclopedia of Genes and Genomes (KEGG) pathway enrichment analysis were conducted with the analyzed DEGs. At last, single-sample gene set enrichment analysis (ssGSEA), CIBERSORT, MCP-COUNTER, and Estimation of STromal and Immune cells in MAlignant Tumor tissues using Expression data (ESTIMATE) algorithms were utilized to discover the relationship between the risk model and the TME. Consequently, the m7G-related lncRNA risk model for COAD patients could be a viable prognostic tool and treatment target.

## Introduction

Colon carcinoma is a frequently diagnosed malignant tumor worldwide, accounting for 10% of all cancer cases worldwide. Colon cancer ranks third among all carcinomas in terms of mortality, and the incidence ratio ranked third in all carcinomas (1). According to world epidemiological data, approximately 1.9 million new cases of colorectal cancer were diagnosed, with 935,000 deaths, in the proportion of around 1/10 of all (2). In China, the incidence rate and mortality rate of colon cancer have been increasing in recent decades. In recent years, great improvements have been made in surgical techniques, chemotherapy, and molecular targeted therapy, leading to an increased survival rate in patients with localized colon cancer (3). Surgery remains to be considered the main treatment modality for those diagnosed with early colon carcinoma (stage I and II) (4); surgery, neoadjuvant radiotherapy, and adjuvant chemotherapy are mainly for those with stage III/IV or stage II which is high risk (5). However, colorectal mortality and the number of deaths from colon cancer per year are still high (6), the potential molecular mechanisms of colon cancer has not been clear (7), and molecular biomarkers for evaluating the survival of this cancer and risk models for evaluating prognosis are still lacking. Therefore, it is imperative to develop a novel model for evaluating the prognosis of patients with colon cancer in order to further ameliorate their prognosis (8).

N5-Methylcytidine (m5C), N6-methyladenosine (m6A), and m7G are some of the common RNA modifications (9). Studies on m5C and m6A research are relatively more than those on m7G, with detailed research on their mechanisms. In recent years, a number of studies on m7G have gradually increased, thus making m7G modifications the next research hotspot of RNA modification. According to extant literature findings (10), tRNA guanine N7 methyltransferase, which belongs to the S-adenosylmethionine (SAM)-dependent RNA methyltransferase family, catalyzes m7G modification. In addition, m7G is also involved in many RNA metabolic processes in the human body, including transcription, mRNA splicing, and translation (11).

As is known to all, long non-encoding RNA (lncRNA) modulates gene transcription and posttranscriptional modification, whose expression is important in human carcinogenesis. According to the recent report, several cancer-related lncRNAs, such as lncRNA MALAT1 in prostate cancer, lncRNA HOTAIR and lncRNA ANRI (12)in cervical cancer, and lncRNA zinc finger protein (ZNF) in gastric cancer, have recently been discovered and their biological involvement in carcinogenesis verified (13). At present, m7G-related lncRNA has not been reported in the literature. Moreover, lncRNA expression is often maladjusted in various cancers and can predict prognosis (14). Therefore, we constructed an m7G-related lncRNA model and investigated its correlation with colon cancer prognosis.

The components of the TME are important for tumor development and metastasis (15). Adipocytes, fibroblasts, immunological cells, tumor-associated macrophages (TAMs), and muscle endothelial cells are primarily found in the TME, all of which mediate paracrine signals to the surrounding (16). The TME, which affects tumor growth, includes immune cells (17). The tumor immune microenvironment (TIME) plays an essential role in tumor-immune interaction which could respond to treatment directly (18). Tumor-infiltrating immune cells are also significant in tumor growth, in immunotherapy response, and in predicting patient survival (19).

**Figure 1:**
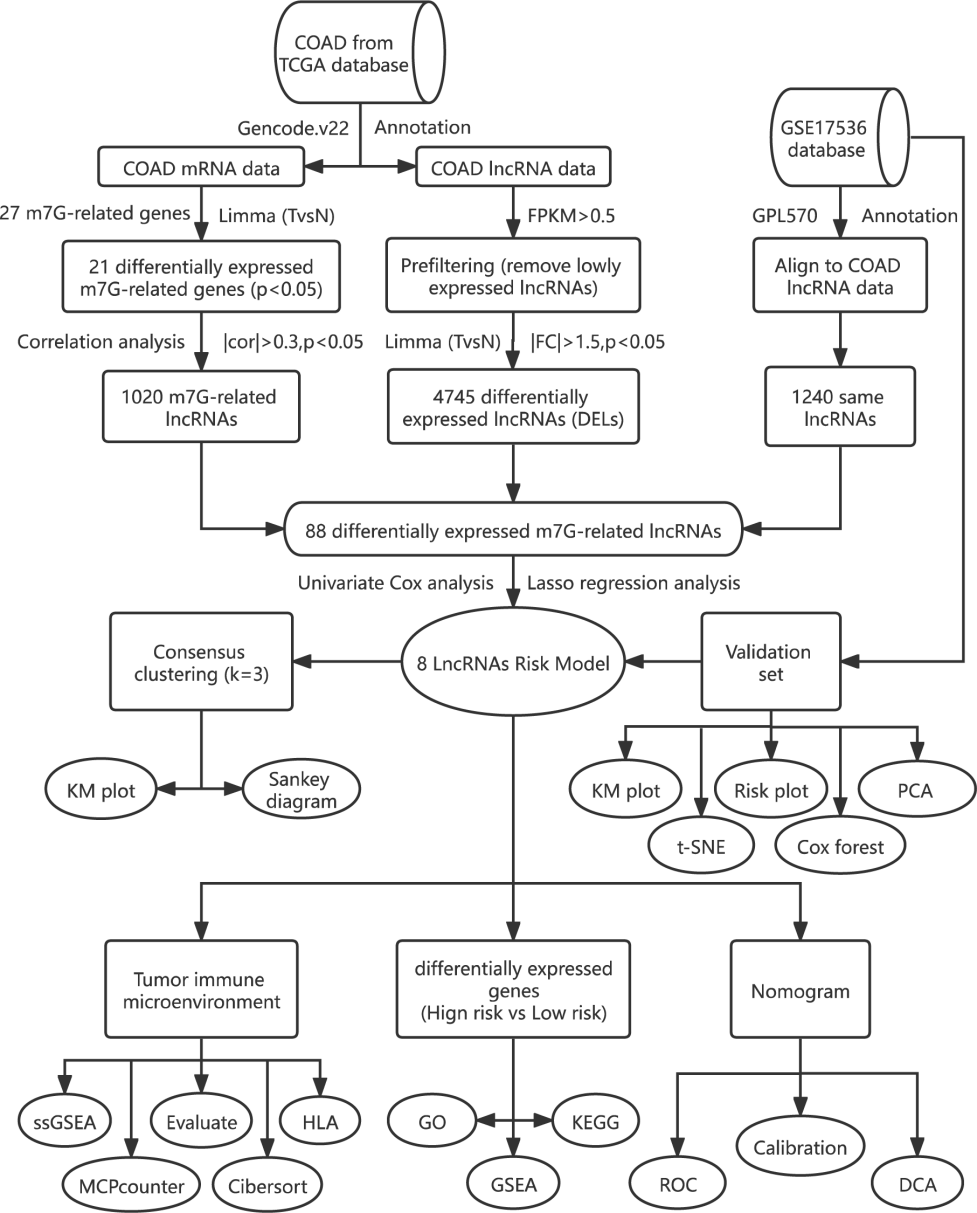
The flowchart of the overall study design.

In our study, we built and validated an eight-m7G-related-lncRNA prognostic risk model. The prognosis of patients was predicted *via* the Kaplan–Meier (KM) chart, receiver operating characteristic (ROC) curve, univariate and multivariate Cox analysis, and nomogram. Through the enrichment analysis of high and low risks, the relevant functions and pathways are obtained. Lastly, we investigated the association with both risk score and immune infiltration using the results of enrichment analysis. The flowchart is displayed in **Figure 1**.

## Materials and methods

### Data collection and analysis

Information of 459 patients was obtained on TCGA website (https://portal.gdc.cancer.gov/repository). Then, we used Perl software to reannotate the Ensemble Genes ID by aligning to gencode.v22 (www.gencodegenes.org/human/release_22.html). A total of 19,712 mRNAs and 14,805 lncRNAs were identified in the COAD dataset. Twenty-seven m7G-related genes were gained by searching the keyword “7-methylguanosine” on the official website of GSEA (http://www.gsea-msigdb.org/gsea/msigdb/search.jsp) and referring to a relevant review (20). Then, using R language software, the expression matrix of m7G-related genes was obtained, and the differential expression analysis was carried out with limma package to obtain 21 meaningful (P < 0.05) m7G-related DEGs. Finally, the visualization results of box diagram, vioplot, and heat diagram were made using the R software package (pheatmap, reshape2, ggpubr, vioplot).

### Construction of a protein–protein interaction network

The protein–protein interaction (PPI) network was created utilizing the online STRING website (cn.string-db.org). The 21 m7G-related DEGs were input into the gene list, homo sapiens were selected, and the medium confidence was set to 0.400. Then, we hid the disconnected nodes of the network and adjusted the position of each node. The barplot package was used to visualize the number of node connection genes, and the counts of connections ≥8 were defined as the hub gene.

### Differentially expressed M7G-related lncRNAs

The lncRNAs gained from the COAD database were filtered out, and the differential expression analysis was carried out with the limma package, obtaining 4,745 m7G-related differentially expressed lncRNAs with significance (P < 0.05, | FC | > 1.5). Through the correlation analysis between m7G-related differential genes and the lncRNA expression level in COAD samples, the lncRNAs related to m7G were identified. Based on the correlation coefficient >0.30 as well as P < 0.05, 1,020 lncRNAs related to m7G were identified. Then, we downloaded the GSE17536 database on the official website of GEO (www.ncbi.nlm.nih. gov/geo/), obtaining the expression matrix and clinical information with the R software package (GEOquery). Reannotation with the GPL570 annotation file was performed to obtain the expression matrix of each symbol. Intersection with the lncRNAs annotated by TCGA was done, finding 1,240 identical lncRNAs. Finally, 88 differentially expressed m7G-related lncRNAs were obtained by intersecting the three types of lncRNAs.

### Construction of the prognostic m7G-related lncRNA risk model

Univariate Cox analysis was performed to obtain 11 prognosis-related lncRNAs (P < 0.05) from these 88 differentially expressed m7G-related lncRNAs in order to check their prognostic significance. In addition, through 1,000-fold cross-validation, the Lasso Cox regression method was used to identify the ideal penalty parameter lambda and the relevant coefficient criterion based on the minimal criterion. Thus, an eight-lncRNA prognostic risk model was built. Then, we obtained the risk score by the following formula: risk score = coef (lncRNAn)*expr (lncRNAn). In the COAD database and GSE17536 dataset, this method was used to compute the risk score of each patient.

### Cluster analysis

Cluster analysis was performed on 452 samples of the COAD data set based on eight lncRNAs in the prognosis model using “ConensusClusterPlus” software package to determine m7G-related molecular subtypes. The number k of clusters was set to 2 to 10, and the “ConensusClusterPlus” program calculated the average contour width of the common member matrix. KM analysis was plotted to estimate the prognosis among different groups, and log-rank test was utilized.

### Validation of the prognostic risk model

We unified the expression amount, survival time, survival status, risk score, and risk level of the eight lncRNAs in each TCGA sample (n = 452) into a table as the training set. The eight lncRNAs in GSE17536 (n = 177) were used as the test set. The prognostic significance of the training set was verified using the test set. A KM plot was utilized to analyze the risk prognosis, and the log-rank test was performed. Moreover, the ROC curve was also drawn using the timeROC program. Then, we utilized the pheatmap package to draw the risk curve, survival state diagram, and risk heat map. The Rtsne and ggplot2 packages were employed for t-SNE as well as principal component analysis (PCA). Finally, the risk score was combined with the clinical characters (age, gender, TNM stage, and grade) of the two data sets for univariate and multivariate Cox analyses, then visualized it with a forest map.

### Nomogram construction and calibration

We further analyzed the clinical characteristics (age, TNM stage, and risk score) that were meaningful (P < 0.05) by univariate Cox analysis to study their clinical value in predicting patient survival. We applied the “RMS” tool to create a nomogram that predicted the 1-, 3-, and 5-year survival rates of COAD patients. We also plotted the calibration curves in the same calibration chart to assess the accuracy of the nomogram. Finally, the decision curve analysis (DCA) curve was drawn using the ggDCA program, which was also utilized to assess the prediction ability of the nomogram and other clinical parameters.

### Gene set enrichment analysis

The link between risk group and Gene Ontology (GO) was investigated using gene set enrichment analysis (GSEA) after TCGA samples were separated into high- and low-risk score groups. For each analysis, 1,000 gene set permutations were done. The enrichment function was chosen based on the following criteria: the gene collection was enriched and evaluated using the clusterProfiler software with a false discovery rate (FDR) of 0.25 and a NOM p value of 0.05. The top five functions enriched by two groups were visualized with an enrichment lot to obtain multiple GSEA diagrams. After that, differential expression analysis of two groups was performed to find DEGs between the two groups. GO and KEGG with the clusterProfiler package were performed to enrich and analyze the DEGs. Then, utilizing the enrichplot and ggplot2 packages, the enrichment results were shown as a barplot, bubble diagram, chord diagram, and cluster circle diagram.

### Association with immune cells and function

Single-sample GSEA (ssGSEA) was applied to examine the differences in immune cell activity, immunological function, and immune route between two groups in the training set and depicted it with a boxplot. The marker genes of different kinds of immune cells could be found in the previous literature (21). The immune score, stromal score, estimated score, and tumor purity were determined using the ESTIMATE program and visualized using a heat map and violin plot. To acquire the composition of invading immune cells in each sample in the training set, the CIBERSORT package was filtered (P 0.05), and the risk score and immune cells were assessed using Pearson correlation. From COAD expression data, the likely MCPcounter package was run to estimate the abundance of immune and non-immune stromal cells, and a violin diagram was drawn to depict the abundance difference between the two groups.

### RNA extraction and qRT-PCR

In order to further confirm the differential expression of the eight lncRNAs, we extracted RNA from fresh frozen tissues with TRIzol reagent (Takara, Japan) and detected the expression level of the eight lncRNAs by qRT-PCR. The cDNA was produced utilizing the PrimeScript RT Master Mix (Takara, Japan) and the designed primers (RiboBio, China). The related GAPDH mRNA expression was identified as an internal control. We collected 24 pairs of fresh colon cancer and adjacent tissues from the Colorectal Center of Jiangsu Provincial People’s Hospital from 2020 to 2021. 2^−ΔΔ^ CT was used to represent the expression. The primer sequences are displayed in **Supplementary Table 1**. Each PCR reaction was carried out three times.

### Statistical analysis

Statistical analysis was performed using R version 4.1.2. The “WilcoxTest” function in the limma package was used to calculate the difference between two preselected groups or paired samples. The correlation between two parameters was evaluated *via* Pearson correlation analysis. The expression matrices of COAD and GSE17536 were batch corrected with the sva package. The survival package was used for KM, univariate, and multivariate Cox regression analyses to calculate the risk ratio, P value, and risk confidence interval. The P value of KM survival curves was calculated by the log-rank test. The glmnet package was used to calculate the optimal penalty parameter lambda and the related coefficient criterion of the Lasso Cox regression algorithm. P < 0.05 was regarded as statistical significance.

## Results

### Identification of differentially expressed m7G-related lncRNAs in COAD patients

**Figure 2:**
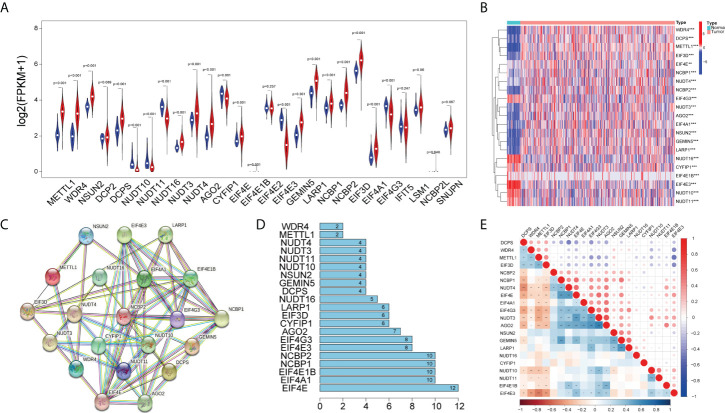
Differentially expressed m7G-related genes. **(A)** Violin plot showing the differential expression of 27 m7G-related genes between tumor and normal tissues from the COAD. **(B)** Heat map of 21 differentially expressed m7G-related genes between tumor and normal tissues (P < 0.05).The PPI network **(C)** and number of interaction nodes **(D)** of 21 differentially expressed m7G-related genes. **(E)** Pearson correlation analysis of 21 differentially expressed m7G-related genes. The red color represents a positive correlation; the blue color represents a negative correlation. *P < 0.05, **P < 0.01, and ***P < 0.001.

**Figure 3:**
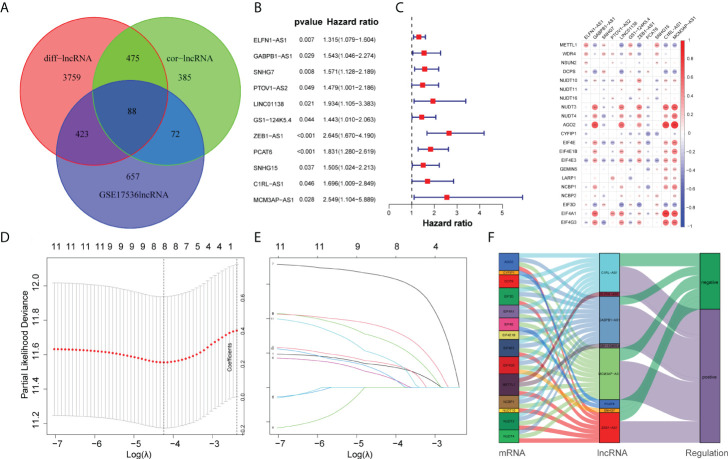
Construction of the prognostic m7G-related lncRNAs risk model. **(A)** Venn diagram of cor-lncRNA, diff-lncRNA, and GSE17536lncRNA. **(B)** Forest map of 11 prognostic m7G-related lncRNAs by univariate Cox analysis (P < 0.05). **(C)** The correlation between 21 differentially expressed m7G-related genes and 11 prognostic m7G-related lncRNAs. The red color represents a positive correlation; the blue color represents a negative correlation. *P < 0.05, **P < 0.01, and ***P < 0.001. **(D)** 1,000 cross-validation to determine the optimal penalty parameter lambda (λ). **(E)** Lasso regression of the 11 m7G-related lncRNAs. **(F)** The Sankey diagram displayed the relationship between the m7G regulators mRNA expression and the m7G-related lncRNAs.

Initially, we obtained 27 m7G-related genes from the official website of GSEA and previous reviews. Combined with the mRNA expression matrix of TCGA, we obtained the expression of these 27 genes in 521 COAD samples. There were 41 paracancerous samples and 480 tumor samples in 521 samples. The difference in expression between tumor samples and normal samples was evaluated. The Wilcox test (P < 0.05) was used to test. We found that among the 27 m7G-related genes, the expressions of METTL1, WDR4, NSUN2, DCPS, NUDT3, NUDT4, AGO2, EIF4E, EIF4E1B, GEMIN5, LARP1, NCBP1, NCBP2, EIF3D, and EIF4A1 increased, the expressions of NUDT10, NUDT11, NUDT16, CYPIP1, EIF4E3, and EIF4G3 decreased, and there was no difference in the expressions of DCP2, EIF4E2, IFIT5, LSM1, NCBP2L, and SNUPN. Then we visualized the 27 genes with a violin map (**Figure 2A**) and made a heat map of 21 differentially expressed genes (**Figure 2B**). Then we used the online website STRING, input the 21 genes into the gene list, and selected *Homo sapiens*. The medium confidence was set to 0.400. The disconnected nodes of the network were hidden, and the position of each node was adjusted. Then we exported a PPI network diagram (**Figure 2C**) and TSV file. We utilized the barplot package to visualize the number of node connection genes (**Figure 2D**) and defined the genes whose counts of connections ≥8 as hub genes. The following hub genes were EIF4E, EIF4A1, EIF4E1B, NCBP1, NCBP2, EIF4E3, and EIF4G3. We also visualized the expression of 21 genes with the corrplot package (**Figure 2E**). Moreover, we removed the normal samples from the lncRNA expression matrix, extracted lncRNAs whose expression was >0.5, and then conducted Pearson correlation analysis based on these 21 genes to screen (|cor| > 0.3, P < 0.05) 1,020 m7G-related lncRNAs. Additionally, 14,805 lncRNAs of TCGA were analyzed for differential expression based on tumor samples and adjacent samples (P < 0.05, | FC | > 1.5). We also obtained the expression matrix of GSE17536 and found 1,240 same lncRNAs as TCGA. Finally, 1,020 m7G-related lncRNAs (cor-lncRNA), 4,745 differentially expressed lncRNAs (diff-lncRNA), and 1,240 identical lncRNAs (GSE17536 lncRNA) were intersected to obtain 88 differentially expressed m7G-related lncRNAs, which were visualized by a Venn diagram (**Figure 3A**).

### Construction of a risk model for COAD patients

Initially, we obtained 27 m7G-related genes from the official website of GSEA and previous reviews. Combined with the mRNA expression matrix of TCGA, we obtained the expression of these 27 genes in 521 COAD samples. There were 41 paracancerous samples and 480 tumor samples in 521 samples. The differential expression between tumor samples and normal samples was analyzed and tested by Wilcox test (P < 0.05). We found that among the 27 m7G-related genes, the expressions of METTL1, WDR4, NSUN2, DCPS, NUDT3, NUDT4, AGO2, EIF4E, EIF4E1B, GEMIN5, LARP1, NCBP1, NCBP2, EIF3D, and EIF4A1 increased, the expressions of NUDT10, NUDT11, NUDT16, CYPIP1, EIF4E3, and EIF4G3 decreased, and there was no difference in the expressions of DCP2, EIF4E2, IFIT5, LSM1, NCBP2L, and SNUPN. Then we visualized the 27 genes with a violin map (**Figure 2A**) and made a heat map of 21 differentially expressed genes (**Figure 2B**). Then we used the online website STRING, input the 21 genes into the gene list, and selected human sapiens. The medium confidence was set to 0.400. The disconnected nodes of the network were hidden, and the position of each node was adjusted. Then we exported a PPI network diagram (**Figure 2C**) and TSV file. We utilized the barplot package to visualize the number of node connection genes (**Figure 2D**) and defined the genes whose counts of connections ≥8 as hub genes. The hub genes were EIF4E, EIF4A1, EIF4E1B, NCBP1, NCBP2, EIF4E3, and EIF4G3. Firstly, the expression matrices of 1,240 identical lncRNAs of TCGA and GSE17536 were obtained respectively, and then the sva package was used for batch correction. We combined the 88 differentially expressed m7G-related lncRNAs with the batch-corrected database to obtain the expression of these 88 lncRNAs in TCGA and GSE17536. Then, we downloaded the clinical data of TCGA and GSE17536, removed the paracancerous samples, and combined them with the expression samples. Finally, 452 TCGA samples and 177 GSE17536 samples with both clinical data (overall survival time and event) and expression were obtained. To discover the prognostic significance of these 88 differentially expressed m7G-associated lncRNAs, univariate Cox analysis (P < 0.05) was used for obtaining 11 prognosis-related lncRNAs (ELFN1-AS1, GABPB1-AS1, SNHG7, PTOV1-AS2, LINC01138, GS1-124K5.4, ZEB1-AS1, PCAT6, SNHG15, C1RL-AS1, MCM3AP-AS1). The 11 lncRNAs were visualized by a forest map (**Figure 3B**). Additionally, the correlation of 11 lncRNAs and 21 DEGs was analyzed, and the correlation diagram (**Figure 3C**) was drawn with the corrplot package. Moreover, we performed Lasso regression analysis on these 11 lncRNAs (**Figures 3D, E**). We determined the optimal penalty parameter lambda and calculated the corresponding coefficient criterion based on the minimum criterion through 1,000-fold cross-validation. Thus, an eight-lncRNA (ELFN1-AS1, GABPB1-AS1, SNHG7, GS1-124K5.4, ZEB1-AS1, PCAT6, C1RL-AS1, MCM3AP-AS1) prognostic risk model was constructed. The following formula was used to determine the risk score: risk score = (0.1248634928618*ELFN1-AS1 expression) + (0.138884459768606*GABPB1-AS1 expression)+(0.271466216016284*SNHG7 expression) + (0.0620449890746169*GS1-124K5.4 expression)+(0.643398387399806*ZEB1-AS1 expression) + (0.344100469251062*PCAT6 expression)+(0.0756308955064826*C1RL-AS1 expression) + (0.170192664397879 *MCM3AP-AS1 expression). Then we analyzed the correlation of the eight lncRNAs and made the correlation circle diagram (**Supplementary Figure 1**). Meanwhile, a differential expression box plot was made in combination with the lncRNA expression matrix of TCGA (**Supplementary Figure 2A**). Furthermore, the expression levels of these eight lncRNAs in 24 frozen paired tissues were tested by qRT-PCR. It was found that they were upregulated in different degrees in tumor tissues (**Supplementary Figure 2B**). Lastly, the correlation between the eight lncRNAs and the target genes was represented by a Sankey diagram (**Figure 3F**). Positive stands for positive correlation and negative stands for negative correlation.

### Identification of m7G-associated clusters and prognostic analysis between clusters

**Figure 4:**
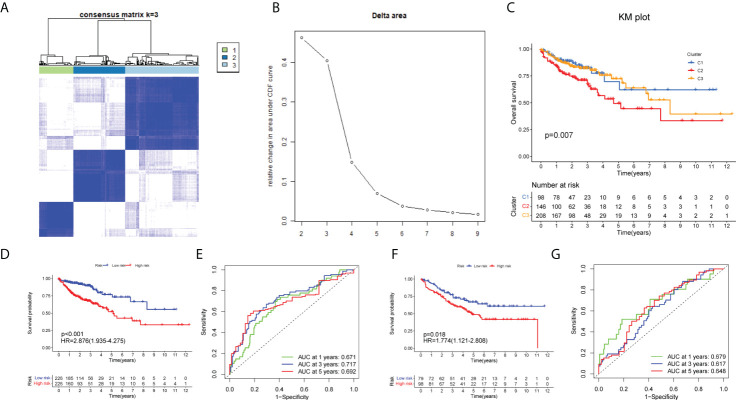
Identification of m7G-associated clusters and prognostic analysis between clusters. **(A)** The consensus matrix (k = 3) of 452 COAD samples by Consensus Cluster analysis. **(B)** The relative change in area under the CDF curve for k = 2–10. The KM plot showing overall survival in three clusters **(C)**, training set **(D)**, and test set **(F)**.The ROC curve of the training set **(D)** and test set **(F)**. The 1-, 3-, and 5-year ROC analyses of risk score in the training set **(E)** and test set **(G)**.

Firstly, we made a further cluster analysis of eight lncRNAs based on the risk model. Cluster analysis was performed on 452 samples of the COAD data set using the “ConsensusClusterPlus” package to determine m7G-associated molecular subtypes. The number k of clusters was selected from 2 to 10 (**Figure 4B**), and the “ConsensusClusterPlus” program calculated the average contour width of the matrix (**Figure 4A**). After careful selection, the best K value was 3 and the samples were divided into three clusters. At last, we analyzed the survival of three clusters and plotted the KM curve (**Figure 4C**). The corresponding p value obtained by the log-rank test was 0.007. There were significant differences in survival among the three subgroups. with cluster1 having a worse prognosis than clusters 2 and 3.

### Validation using the GSE17536 set

**Figure 5:**
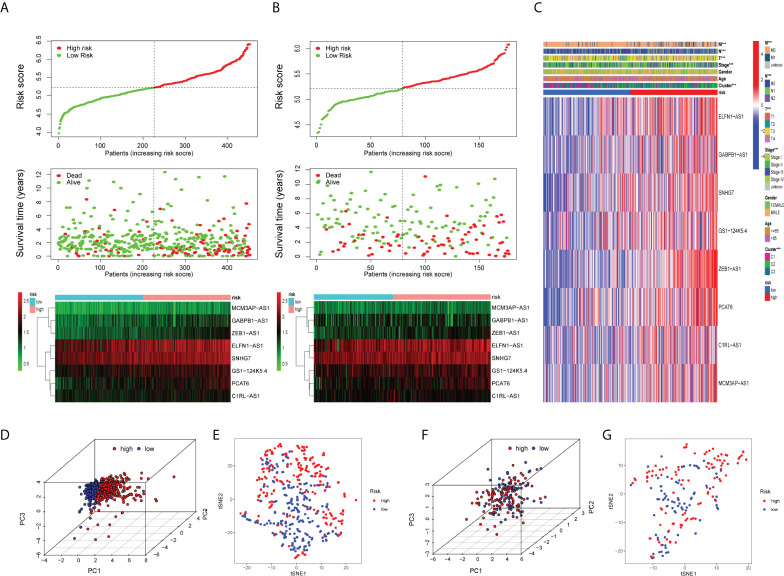
Validation of the prognostic risk model. Scatter plot revealing the risk score distribution of high risk and low risk and the relationship between survival time and risk score based on the training set **(A)** and test set **(B)**. Heat map displaying the differential expression of the eight prognostic m7G-related lncRNAs in the high- or low-risk group. **(C)** Heat map showing clinicopathological features (TMN stage, stage, age, gender) and differences in the expression of eight lncRNAs in the high- and low-risk groups. *P < 0.05, **P < 0.01, and ***P < 0.001. The 3D scatter plot of PCA results of the training set **(D)** and test set **(F)**.The t-SNE analysis of the training set **(E)** and test set **(G)**.

**Figure 6:**
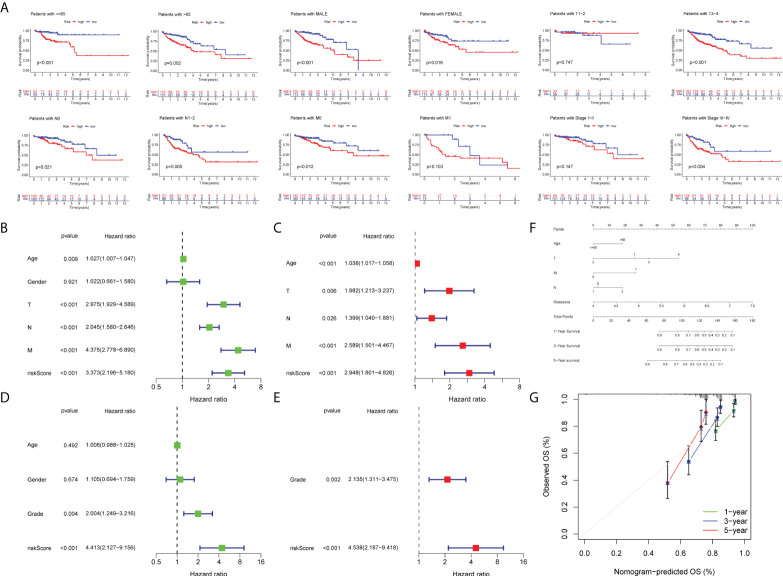
Survival analysis and construction of a nomogram. **(A)** Survival analysis in subgroups including gender, age, and tumor stages. Univariate Cox regression analysis revealing the association between patients’ overall survival and clinicopathological parameters along with m7G-related lncRNA risk scores in the training set **(B)** and test set **(D)**. Multivariate Cox regression analysis uncovering independent prognostic factors in the training set **(C)** and test set **(E)**. **(F)** Nomogram depending on the m7G-related lncRNA risk score and other clinicopathologic feature predicting the 1-, 3-, and 5-year overall survival for COAD patients. **(G)** Calibration curves illustrating the consistency between predicted and observed 1-, 3-, and 5-year overall survival rates in COAD patients based on the nomogram.

Initially, we identified TCGA database (n = 452) as the training set and the GSE17536 database as the test set for verification. We plotted the KM curve (**Figures 4D, F**) and ROC curve (**Figures 4E, G**) of the training set and the test set. The P value of the training set (P < 0.001) and test set (P = 0.018) was obtained by the log-rank test. The HR of the training set was 2.876 and 95% CI: 1.935–4.275, while the HR of the test set was 1.774 and 95% CI: 1.121–2.808. The risk score’s area under the ROC curve (AUC) value was examined to determine its specificity and sensitivity in predicting patient prognosis in the two data sets. In the training set, the AUC values for the 1-, 3-, and 5-year risk scores were 0.671, 0717, and 0.692, whereas the AUC values in the test set were 0.679, 0.617, and 0.648. The risk score and survival status of COAD patients (**Figure 5A**) and GSE17536 patients (**Figure 5B**) were displayed using a risk curve, scatter plot, and risk heat map. We also made an expression heat map of clinicopathological features (TMN stage, stage, age, gender), clusters, and risk score based on TCGA database (**Figure 5C**). Additionally, the Rtsne package and ggplot2 package were used for t-SNE analysis of the training set (**Figure 5E**)and test set (**Figure 5G**). The scatterplot3d package was used to make 3D images of PCA analysis of the training set (**Figure 5D**) and test set (**Figure 5F**). It was demonstrated that the two groups of the training set and test set were heterogeneous. Moreover, we further subdivided each clinicopathological feature (TMN stage, stage, age, gender) and analyzed the survival of the risk scores of each subgroup (**Figure 6A**). The KM curve showed that the subgroups with significant survival (P < 0.05) in two groups are the younger (age less than 65)or older (age greater than 65)patients, male or female, stage III–IV groups, T III–IV groups, N0 or N I–II groups, and M0 (patients without any metastasis) groups. Subsequently, we utilized univariate and multivariate Cox regression analyses to see if the risk scores obtained by the two risk models may well be employed as an independent COAD prognostic signature. Univariate Cox regression analysis showed that age (HR: 1.027, 95% CI: 1.007–1.047, P = 0.009), T stage (HR: 2.975, 95% CI: 1.929–4.589, P < 0.001), N stage (HR: 2.045, 95% CI: 1.580–2.646, P < 0.001), M stage (HR: 4.375, 95% CI: 2.778–6.890, P < 0.001), and the risk score (HR: 3.373, 95% CI: 2.196–5.180, P < 0.001) in the training set were significantly positively associated with OS (**Figure 6B**). The grade (HR: 2.004, 95% CI: 1.249–3.216, P = 0.004) and risk score (HR: 4.413, 95% CI: 2.127–9.156, P < 0.001) of the test set were significantly positively correlated with OS (**Figure 6D**). Multivariate analysis of significant factors in univariate analysis showed that age (HR: 1.038, 95% CI: 1.017–1.058, P < 0.001), T stage (HR: 1.982, 95% CI: (1.213–3.237, P = 0.006), N stage (HR: 1.399, 95% CI: 1.040–1.881, P < 0.001), M stage (HR: 2.589, 95% CI: 1.501–4.467, P < 0.001), and risk score (HR: 2.948, 95% CI: 1.801–4.826, P < 0.001) were significantly associated with OS in COAD (**Figure 6C**), whereas the grade (HR: 2.135, 95% CI: 1.311–3.475, P = 0.002) and risk score (HR: 4.538, 95% CI: 2.187–9.418, P < 0.001) of the GSE17536 dataset were significantly positively correlated with OS (**Figure 6E**), suggesting that these two parameters can be used as independent prognostic factors. The risk score was found to be a useful independent predictor of outcome, outperforming other clinicopathological characteristics such as TMN stage, stage, age, sex, and grade.

### Formulation and examination of a nomogram

**Figure 7:**
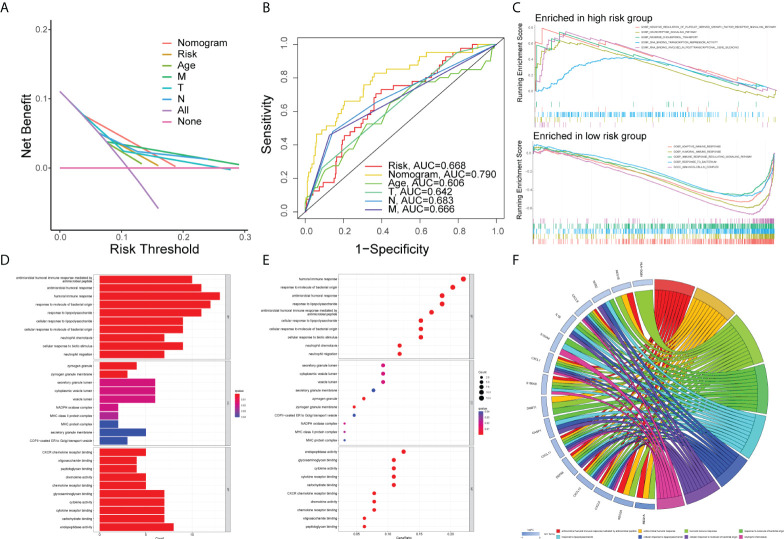
Gene set enrichment analysis. DCA curve **(A)** and ROC curve **(B)** of the nomogram, risk, and other clinicopathologic feature in COAD. **(C)** GSEA results illustrating 10 significant enrichment of GO in low-risk and high-risk groups. The results of GO enrichment analysis of the differentially expressed genes shown by barplot **(D)**, bubble chart **(E)**, and chord diagram **(F)**.

First of all, we created a nomogram comprising clinical characteristics of TMN stage, age, and risk score depending on the outcomes of univariate and multivariate Cox analyses (**Figure 6F**). We obtained the total score of a patient according to his clinical information, which could be used for assessing the prognosis of patients. Next, we also plotted the calibration curves (**Figure 6G**). The higher the number of curves of the three calibration curves close to the standard curve, the more accurate the prediction of the nomogram was. In addition, we performed the DCA (**Figure 7A**). The benefits of the nomogram were much higher than those of the extreme curves, according to the image results. The nomogram curve was higher than other clinical features (age, TNM stage) and risk score curve, indicating that the nomogram was more reliable in predicting survival rate. Finally, we made the ROC curve (**Figure 7B**) of multiple clinical factors (age, TNM stage, risk, and nomogram) and calculated the area under the ROC curve (AUC). We found that the AUC values of risk, nomogram, age, and TNM stage were 0.668, 0790, 0.606, 0.642, 0.683, and 0.666, respectively. When the AUC values of the nomogram and other clinical factors were compared, the nomogram was found to have a significantly higher AUC value, implying that the nomogram was a good prognostic predictor.

### Gene set enrichment analysis

First of all, the COAD samples (n = 452) were divided into high- and low-risk groups. The connection between GO and risk group was investigated using GSEA. The following conditions were used to filter the enrichment function: NOM P < 0.05, FDR < 0.25. The top five functions enriched in the high-risk group were GOBP_NEGATIVE_REGULATION_OF_PLATELET_DERIVED_GROWTH_FACTOR_RECEPTOR_SIGNALING_PATHWAY, GOBP_NEUROPEPTIDE_SIGNALING_PATHWAY, GOBP_REVERSE_CHOLESTEROL_TRANSPORT, GOMF_DNA_BINDING_TRANSCRIPTION_REPRESSOR_ACTIVITY, and GOMF_RNA_BINDING_INVOLVED_IN_POSTTRANSCRIPTIONAL_GENE_SILENCING, and those enriched in the low-risk group were GOBP_ADAPTIVE_IMMUNE_RESPONSE, GOBP_HUMORAL_IMMUNE_RESPONSE, GOBP_IMMUNE_RESPONSE_REGULATING_SIGNALING_PATHWAY, GOBP_RESPONSE_TO_BACTERIUM, and GOCC_IMMUNOGLOBULIN_COMPLEX groups which were visualized with an enrichment lot to obtain multiple GSEA diagrams (**Figure 7C**). The high-risk group was shown to be mostly linked to DNA transcription and RNA posttranscriptional modification, while the low-risk group was mostly linked to immunological infiltration. Next, we used the sva package to batch correct the same 16,397 mRNAs of TCGA and GSE17536, obtaining the corrected expression matrix. Then we used differential expression analysis to find 67 genes that were differentially expressed between the two groups (P<0.05, | FC | > 1.5). Then, we ran GO and KEGG enrichment analyses. The top 10 molecular functions (MF), biological process (BP), and cellular components (CC) according to their enrichment score were visualized by the barplot (**Figure 7D**), bubble diagram (**Figure 7E**), and chord diagram (**Figure 7F**). The DEGs were mainly enriched in “antimicrobial humoral immune response mediated by antimicrobial peptide”, “antimicrobial humoral response”, “humoral immune response”, “response to molecule of bacterial origin”, “response to lipopolysaccharide”, “cellular response to lipopolysaccharide”, “cellular response to molecule of bacterial origin”, “neutrophil chemotaxis”, and other functions by differentially expressed genes. The top 30 KEGG pathways were visualized by the barplot (**Supplementary Figure 3A**), bubble diagram (**Supplementary Figure 3B**), and cluster circle diagram (**Supplementary Figure 3C**). The DEGs enriched in “IL-17 signaling pathway”, “rheumatoid arthritis”, “viral protein interaction with cytokine and cytokine receptor”, “toll-like receptor signaling pathway”, “influenza A”, “legionellosis”, “cytokine-cytokine receptor interaction” and “pertussis” were mainly activated.

### Immune infiltration analysis of the risk model

**Figure 8:**
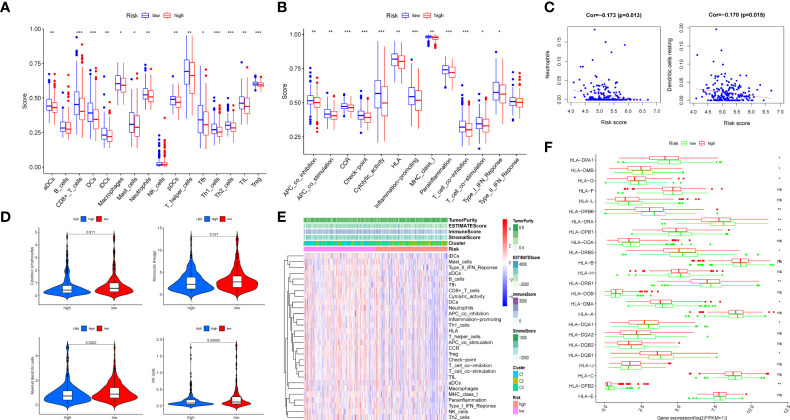
Immune infiltration analysis of the prognostic m7G-related lncRNA risk model. The infiltrating levels of 16 immune cell types **(A)** and 13 immune functions **(B)** in high-risk and low-risk groups estimated by ssGSEA. **(C)** The correlation of immune score and risk score calculated by CIBERSORT. **(D)** The violin diagram revealing the abundance of 10 types of immune and stromal cells between two groups *via* MCPcounter. **(E)** Heat map of 29 immune cells and functions displaying the difference of the immune score, stromal score, estimated score, and tumor purity in two groups through ESTIMATE. **(F)** Box plot of 24 MHC molecules’ expression level in two groups. ns, not significant, *P < 0.05, **P < 0.01, and ***P < 0.001.

The results of the GSEA showed that the low-risk category was mostly associated with immunological infiltration, according to GSEA outcomes. As a result, we discovered the association in risk score and the immune infiltration microenvironment. To begin, the infiltration of 16 immune cells and the scores of 13 immunological functions were analyzed utilizing the ssGSEA method. In comparison to the high-risk group, the low-risk group had stronger immune cell infiltration (**Figure 8A**) and more immune-related functions or pathways (**Figure 8B**). Secondly, COAD samples with a CIBERSORT output p value less than 0.05 were screened using the CIBERSORT algorithm for research. A bar graph was used for illustrating the percentage of 22 immune cells in 220 samples (**Supplementary Figure 4**). Only neutrophils and dendritic cells resting were negatively connected (P < 0.05) in the correlation analysis between these 22 immune cells and risk score (**Figure 8C**). In addition, based on COAD expression data, the MCPcounter software was applied to calculate the content of 10 categories of immune and stromal cells, and the violin diagram (**Figure 8D**) was created to demonstrate the abundance difference between the two groups. The low-risk group had considerably more cytotoxic lymphocytes, monocytic lineage, myeloid dendritic cells, and natural killer cells (NK cells) than the high-risk group (P < 0.05). Moreover, we obtained the immune score, stromal score, estimated score, and tumor purity of every patient using the ESTIMATE algorithm according to the proportion of immune and stromal cells in the TME. Then, based on the immune score, interstitial score, estimated score, tumor purity, cluster, and risk group, the score heat map of 29 immune cells and functions obtained by ssGSEA was made (**Figure 8E**). Furthermore, we detected the expression differences of 24 major histocompatibility complex (MHC) molecules in two groups and visualized them with a box diagram (**Figure 8F**). We discovered that the expression in the low-risk group was significantly greater than in the high-risk group. Finally, we examined the expression differences of 10 common immune checkpoint molecules (PDCD1, CD274, PDCD1LG2, CTLA4, LAG3, SIGLEC7, HAVCR2, LILRB2, VSIR, and FCGR3A) in two groups and made a box plot (**Supplementary Figure 5**). We discovered that CD274, PDCD1LG2, LAG3, SIGLEC7, HAVCR2, LILRB2, and FCGR3A were significantly overexpressed in the low-risk group.

## Discussion

As everyone knows, colon cancer is one of the most common digestive tract carcinomas, with high malignancy and invasiveness, as well as a high incidence and fatality rate (22). As a result, researching prognostic markers in colon cancer is crucial (23). lncRNA is also important in the genesis and progression of colon cancer. For instance, the lncRNA MALAT1, which is upregulated in colon carcinoma, may accelerate colon cancer cell growth (24). CCAT1 and CCAT2 have also been reported to be closely related to colorectal cancer (25). According to the existing literature reports, the number of prognostic models constructed by using the public database COAD is increasing. For instance, m6A-associated lncRNAs are potential prognostic biomarkers of colon cancer (26), prognostic risk model of pyroptosis-associated lncRNAs (27), and establishment and validation of the ferroptosis-related lncRNA prognostic signature (28). In addition, RNA modification is involved in the biosynthesis, metabolism, and structural stability of RNA molecules, which is highly related to tumors (29). Overall, this is the first study in colon cancer to develop an m7G-related lncRNA risk model for predicting patient prognosis. Furthermore, this prognostic model is strongly correlated with clinicopathological factors, immune cells, and immune-related functions. Consequently, it could be utilized to guide immune targeted therapy and predict patient survival.

In our research, the prognostic model we constructed included eight lncRNAs, namely, ELFN1-AS1, GABPB1-AS1, SNHG7, GS1-124K5.4, ZEB1-AS1, PCAT6, C1RL-AS1, and MCM3AP-AS1. ELFN1-AS1 may improve colon cancer cell growth and migration while activating ERK and the epithelial–mesenchymal transition (EMT) pathway (30). Small nuclear RNA host gene 7 (SNHG7) is highly expressed in gastric and thyroid cancer and is associated with tumor stage and overall survival (31, 32). By overexpressing zinc finger enhancer-binding protein (ZEB1), ZEB1-AS1 was able to accelerate osteosarcoma and prostate cancer progression (33, 34). In addition, prostate cancer-associated transcript 6 (PCAT6) could promote the oncogenesis and angiogenesis of triple-negative breast cancer by regulating VEGFR2 (35). AKT/β-catenin/c-Myc pathway was activated by C1RL-AS1 to promote the cancerous behavior in stomach adenocarcinoma cells (36). Moreover, by affecting the miR-194-5p/FOXA1 axis, MCM3AP-AS1 has been shown to increase hepatocellular cancer growth (37). However, there are few reports concerning GABPB1-AS1 and GS1-124K5.4 in tumors. In summary, these lncRNAs were critical in the tumorigenesis and progression of tumors. Therefore, using lncRNA to construct our prognostic model seemed feasible and convincing.

Next, based on the GSEA outcomes of two groups, the high-risk group was mainly related to RNA modification, whereas the low-risk group was primarily enriched in immune cells and function. Based on the existing research, we have found some lncRNAs that could be identified as immunomodulatory factors, including lncRNA-COX2, THRIL, lncRNA-EPS, and MORRBID (38). Colon cancer was infiltrated by various immune cells, including tumor-associated macrophages (TAMs), tumor-associated neutrophils (TANs), CD8T cells, and cancer-associated fibroblasts (CAFs) (39). In our study, the risk score of lncRNAs was associated with immune cells including neutrophils, resting dendritic cells, cytotoxic lymphocytes, monocytic lineage, myeloid dendritic cells, and NK cells. Their abundance was much greater in the low-risk group than in the high-risk group. Moreover, we also used R programs like ESTIMATE and ssGSEA to assess the level of immunological infiltration. To summarize, the tumor immune infiltration microenvironment was found to be strongly associated with our risk model.

Of course, our research also have many deficiencies. First of all, the AUC values of the 1-, 3-, and 5-year risk scores of the training set and test set were basically <0.7. The accuracy for prediction was not very high; thus, the risk model needed to be improved. For example, we could set more strict screening criteria of FC value and P value in differential expression analysis. In univariate Cox analysis, the threshold of P could be set to 0.001, filtering out better prognostic lncRNAs. Secondly, the validation training set was only verified by the retrospective data of GEO. We should also verify its long-term clinical value through more prospective studies. Additionally, we also discovered the association among risk score and immune cells, immune function, immune score, and MHC molecules. Referring to a recent study, the greater the tumor mutation load, the worse the prognosis of patients (40). Immune infiltration in tumors was an important prognostic marker of immunotherapeutic response (41). Therefore, it is important to study the relationship between tumor mutation burden and the response of immunotherapy. Last but not least, the eight lncRNAs in the model needed further experimental verification *in vivo* and *in vitro* to test their role in the tumorigenesis and progression of colon cancer.

## Conclusion

According to the transcriptome expression matrix and clinical data of TCGA, we created a prognostic risk model consisting of eight m7G-related lncRNAs for COAD patients. Next, we also verified the prognostic model according to the expression matrix and clinical data of the GSE17536 dataset. This predictive risk model was shown to have independent prognostic significance and could effectively predict the OS rate for COAD patients. Furthermore, our research has provided a deeper understanding of the association between this prognostic model and the tumor-immune microenvironment. Finally, the m7G-related lncRNA risk model may help us identify possible COAD signatures or therapeutic targets.

## Data availability statement

The original contributions presented in the study are included in the article/**Supplementary Material**. Further inquiries can be directed to the corresponding author/s.

## Ethics statement

This study was reviewed and approved by the Ethical Committee of First Affiliated Hospital of Nanjing Medical University. The patients/participants provided their written informed consent to participate in this study.

## Author contributions

SY and JZ designed the study. YS and XW collected and analyzed the data of TCGA and GEO data. SY and ZC performed the experiments. XW validated our method of data processing and statistical analysis. SY and JZ drafted the manuscript. YS secured funding for the study. XW and YS reviewed and supervised the study. All authors contributed to the article and approved the submitted version.

## Funding

This work was supported by the Jiangsu Provincial Natural Science Foundation for Basic Research, China (Grant No. BK20201491)

## Acknowledgments

We would like to thank TCGA and GEO as public databases, providing free data for relevant research. We acknowledge the Core Facility of Jiangsu Provincial People’s Hospital for its help in the detection of experimental samples. We thank Bullet Edits Limited for the linguistic editing and proofreading of the manuscript.

## Conflict of interest

The authors declare that the research was conducted in the absence of any commercial or financial relationships that could be construed as a potential conflict of interest.

## Publisher’s note

All claims expressed in this article are solely those of the authors and do not necessarily represent those of their affiliated organizations, or those of the publisher, the editors and the reviewers. Any product that may be evaluated in this article, or claim that may be made by its manufacturer, is not guaranteed or endorsed by the publisher.
