# Supplementary material for: A novel m7G-related lncRNA risk model for predicting prognosis and evaluating the tumor immune microenvironment in colon carcinoma
Source: Front Oncol. 2022 Aug 4;12:934928. doi: 10.3389/fonc.2022.934928 (PMC9386370; doi:10.3389/fonc.2022.934928)
Supplement: Supplementary file 1 [file DataSheet_1.docx]

Supplementary Material

# Supplementary Figures and Tables

**1.1 Supplementary Tables**

| lncRNA | 5'-3' |
| --- | --- |
| GS1-124K5.4 F | CCGCCGACACCAATAAGCT |
| GS1-124K5.4 R | TTCTGCCTGAACGTGCCCT |
| PCAT6 F | AAACCGCCCTCATTTGTGC |
| PCAT6 R | GGGAGTTGGATGGACCGAAT |
| ELFN1-AS1 F | TCATTCACTCCGAGACGCAG |
| ELFN1-AS1 R | AGAGAAGGGCCAGGATTTAAAC |
| GABPB1-AS1 F | TTTACCAGCGAAGAGATCAACTTC |
| GABPB1-AS1 R | CTGAAGCGCTTTGTGTGTAGC |
| SNHG7 F | CGGGAAGGAGGTGACTTCG |
| SNHG7 R | CTGGTCAGCCTGGTCACTCT |
| ZEB1-AS1 F | CTCAACACCTAGTGGAGTACGTAG |
| ZEB1-AS1 R | CCTTACTGTCAAGAACAGGGTCAG |
| C1RL-AS1 F | CTTGCTCCAATGAGGACCCA |
| C1RL-AS1 R | GGCTGTTTTCGGAAGAGGCT |
| MCM3AP-AS1 F | CCCTGATTCCACCTAGTGTTCAT |
| MCM3AP-AS1 R | AGCCTAGATGGAAGTCTGAGTGTGT |

## 1.2Supplementary Figures

## **
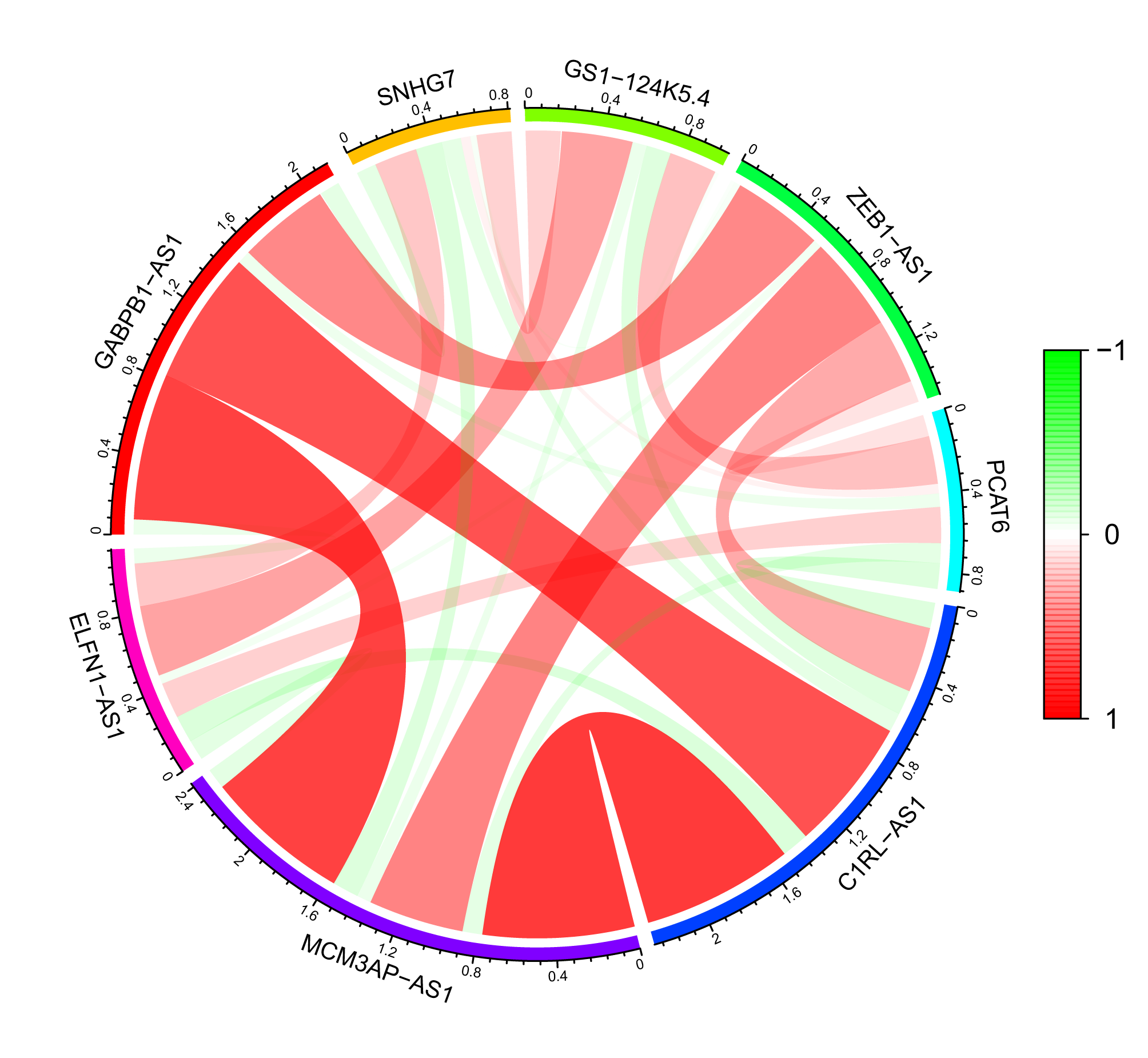
**

## **Supplementary Figure 1.** The correlation circle diagram of the 8 prognostic m7G-related lncRNAs.

**
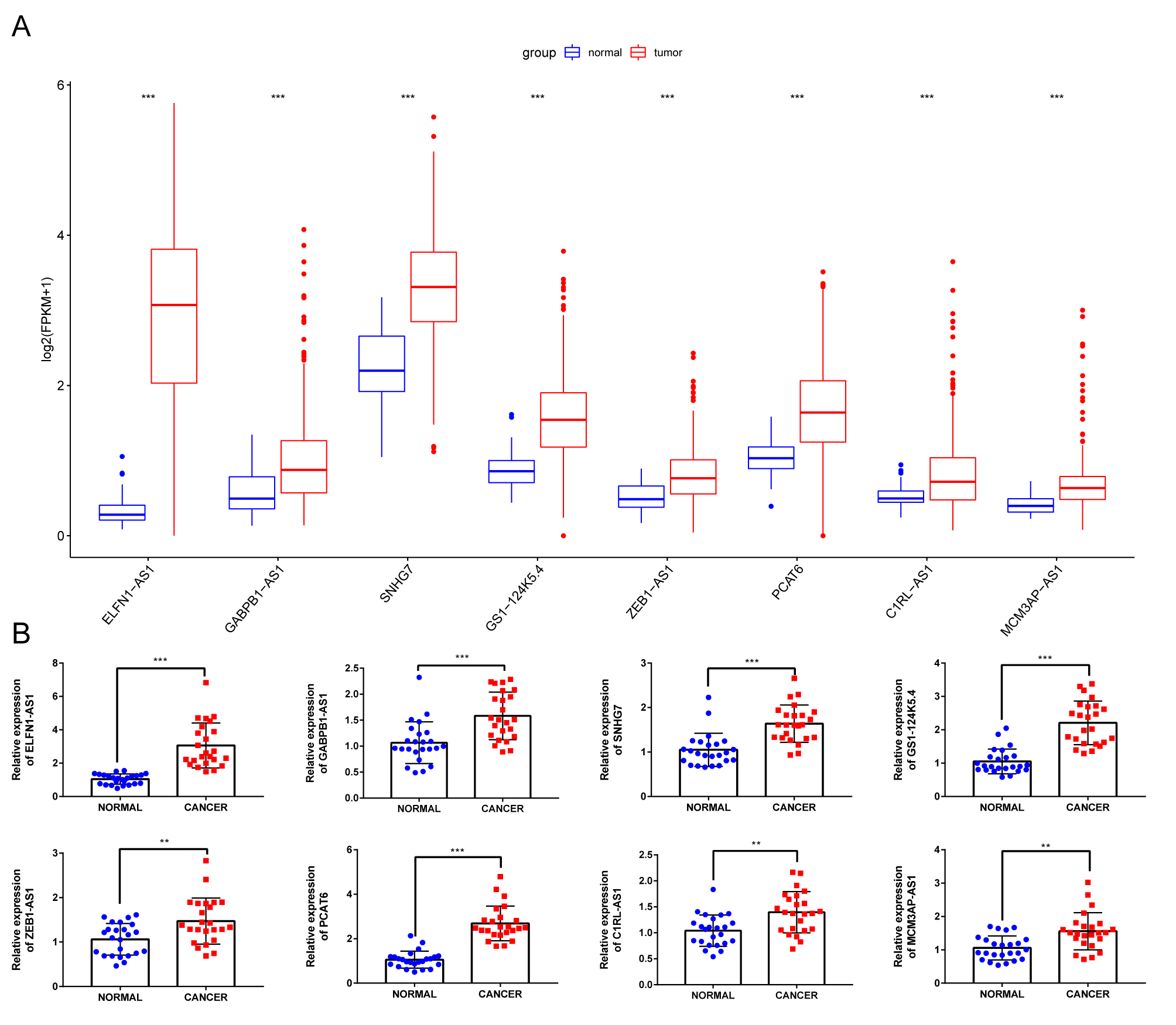
**

**Supplementary Figure 2.** (A)The differential expression box plot of the 8 prognostic m7G-related lncRNAs in COAD.(B) qRT-PCR detecting the relative expression of the 8 m7G-related lncRNAs respectively in 24 paired tissues.

**
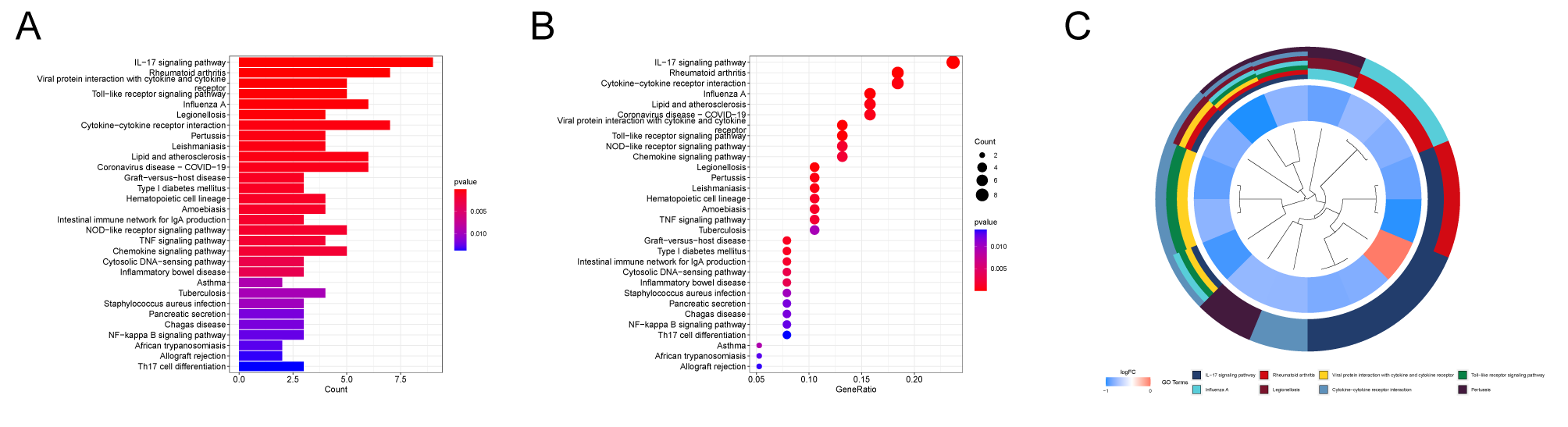
**

**Supplementary Figure 3.** The results of KEGG enrichment analysis of the

differentially expressed genes showing by barplot(A),bubble chart(B) and cluster circle diagram(C).

**
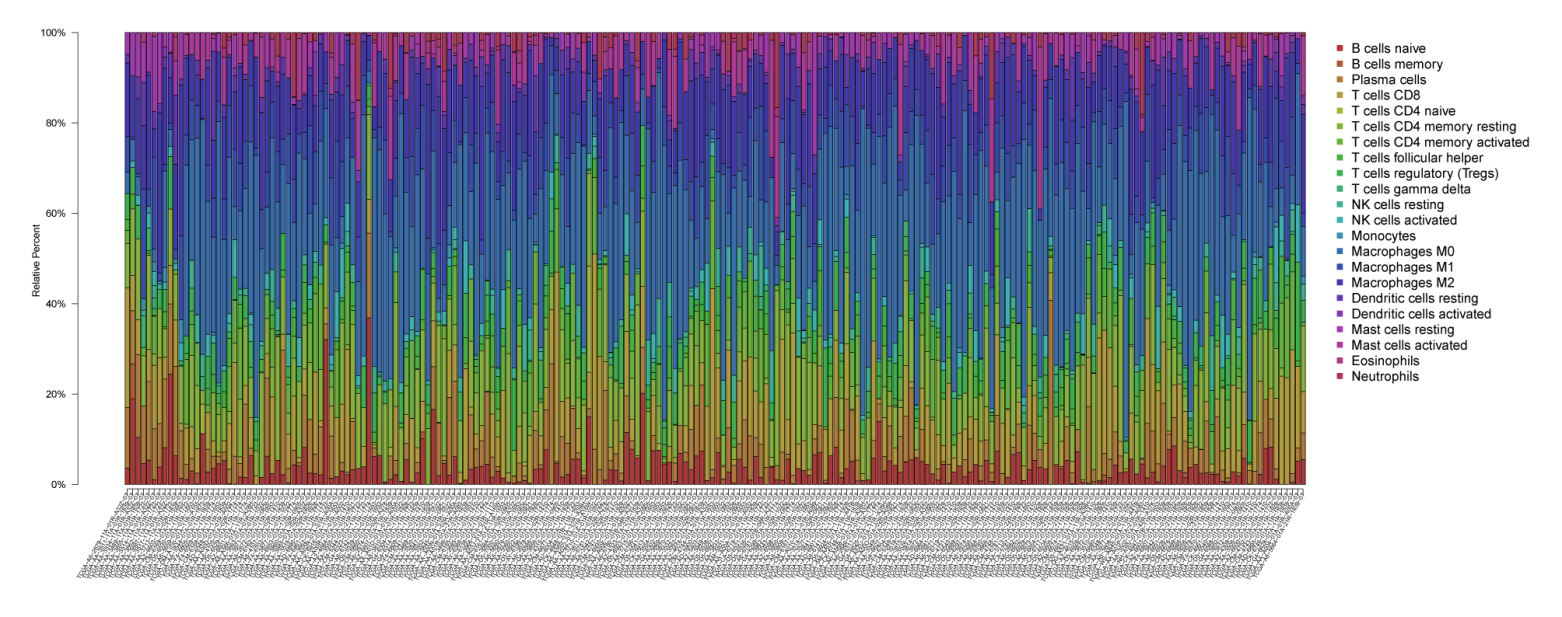
**

**Supplementary Figure 4.** The bar graph displaying the percentage of 22 immune cells in the 220 samples calculated by CIBERSORT.


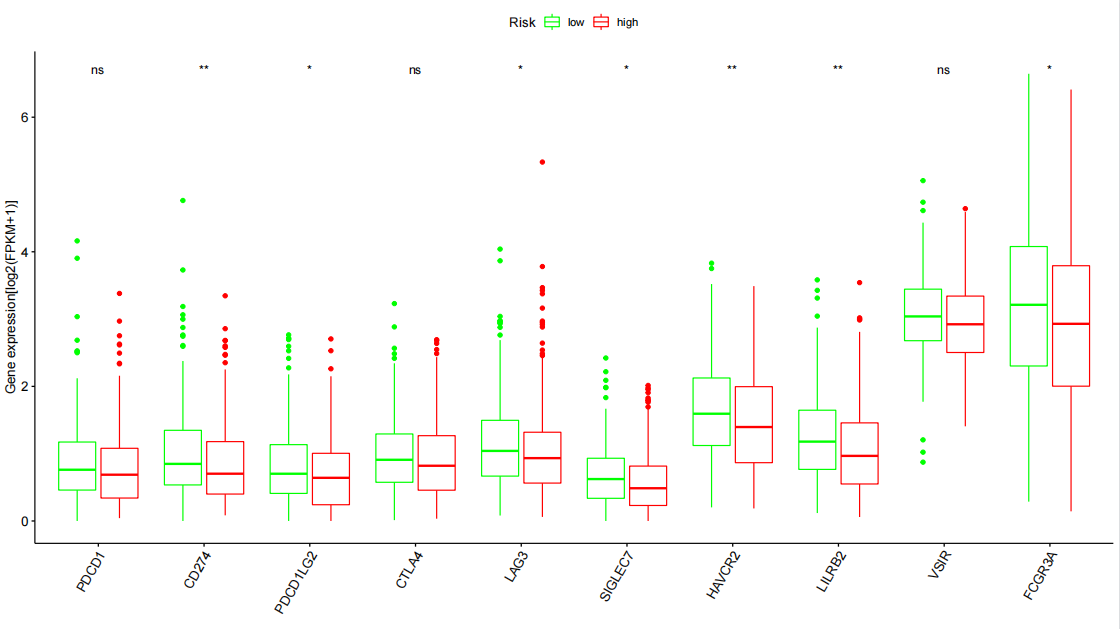


**Supplementary Figure 5.** The expression box plot of 10 common immune checkpoints between low- and high-risk groups.
